# Validation of Blood-Based Biomarkers After Mild Traumatic Brain Injury with GCS 15 in a Singapore Emergency Department: An Observational Study

**DOI:** 10.3390/medicina62061095

**Published:** 2026-06-05

**Authors:** Win Sen Kuan, Ying Wei Yau, Desiree Xin Ying Lim, Chew Kiat Yeoh, Nicole Mun Teng Cheung, Hannah Xin Yi Lim, Mui Teng Chua

**Affiliations:** 1 Emergency Medicine Department, National University Hospital, National University Health System, Singapore 119074, Singapore; ying_wei_yau@nuhs.edu.sg (Y.W.Y.); desiree.lim@mohh.com.sg (D.X.Y.L.); chew_kiat_yeoh@nuhs.edu.sg (C.K.Y.); nicole_cheung@nuhs.edu.sg (N.M.T.C.); hannah.lim@mohh.com.sg (H.X.Y.L.); mui_teng_chua@nuhs.edu.sg (M.T.C.); 2 Department of Surgery, Yong Loo Lin School of Medicine, National University of Singapore, Singapore 119228, Singapore

**Keywords:** mild traumatic brain injury, ubiquitin carboxyl-terminal hydrolase-L1, glial fibrillary acidic protein, intracranial hemorrhage

## Abstract

*Background and Objectives*: Traumatic brain injury (TBI) affects millions of people worldwide. The Glasgow Coma Scale (GCS) is commonly used to characterize its severity. Head computed tomography (CT) is frequently the diagnostic imaging modality of choice. Recently, blood-based biomarkers such as ubiquitin C-terminal hydrolase-L1 (UCH-L1) and glial fibrillary acidic protein (GFAP) have emerged as possible adjuncts to head CT in evaluating mild TBI (mTBI). We aim to validate the performance of the Abbott Alinity i TBI test (UCH-L1 and GFAP) compared to head CT in an Asian cohort with mTBI and GCS 15. *Materials and Methods*: This prospective observational study was conducted at a tertiary academic medical center from 2 December 2024 to 19 March 2025. Patients aged 21 years and above who sustained head injury within 12 h of ED attendance had GCS of 15 and required head CT as per attending physician were eligible. Plasma was separated from whole blood within 10 min of collection and immediately stored at −20 °C. UCH-L1 and GFAP levels were analyzed in batches within 28 days of recruitment at the hospital central laboratory using the Alinity i TBI test. *Results*: Among 120 patients enrolled, there was predominance of males (55.8%, 67/120) and Chinese ethnicity (75.8%, 91/120). The median age was 73 (interquartile range [IQR] 56 to 79) years. Overall incidence of positive head CT was 9.2% (11/120); all 11 patients had positive Alinity i TBI tests. The sensitivity and negative predictive value of the biomarkers in our cohort were both 100% (95% confidence intervals [CIs] 71.5% to 100% and 78.2% to 100%, respectively), specificity 13.8% (95% CI 7.9% to 21.7%) and positive predictive value 10.5% (95%CI 5.4% to 18%). Exploratory post hoc analysis suggested that GFAP alone, at the prespecified assay threshold, was associated with modestly higher specificity [21.1% (95% CI 13.9% to 30.0%)] in this cohort. *Conclusions*: The Alinity i TBI test can safely rule out intracranial injury in patients with mTBI and GCS 15 presenting within 12 h of injury. However, specificity was low, limiting its ability to reduce head CT use in its current form. Exploratory post hoc analyses of the individual biomarkers, particularly GFAP alone, should be interpreted cautiously. Future studies should focus on optimizing specificity while maintaining a high degree of sensitivity.

## 1. Introduction

Traumatic brain injury (TBI), defined as an alteration in brain function or evidence of brain pathology caused by an external force, is a major public health concern resulting in substantial mortality and disability worldwide [1,2]. Up to 60 million people sustain TBI annually, costing US$ 400 million to the global economy [3]. The emergency department (ED) is the main portal of care for patients with TBI [4]. There is scant information on the epidemiology of TBI in Singapore. Extrapolation of data from a tertiary trauma center in 2013 and 2014 estimated 1900 ED attendances for TBI annually, of which almost one-fifth of patients were admitted [5,6]. The incidence of and admissions for TBI among older adults aged 65 years and above are also increasing [7,8].

The Glasgow Coma Scale (GCS) is the most common and widely used scoring system to estimate TBI severity by evaluating motor, verbal, and eye-opening responses on a 15-point scale [9]. This scoring system classifies TBIs as mild (GCS 13–15), moderate (GCS 9–12), or severe (GCS 3–8). Over 90% of TBI patients who present to hospitals have mild TBI (mTBI) [3]. Standard approaches to identifying and assessing the severity of TBI include applying the GCS, performing neuropsychological assessments, and utilizing neuroimaging to determine the damage caused by the injury [10].

Currently, head computed tomography (CT) is the recommended diagnostic imaging modality in evaluating patients for TBI [11]. Despite its use, only about 7% of patients with mTBI who present to the ED show positive traumatic lesions on head CT, and merely 1% will require neurosurgical intervention [12]. This highlights an increasing drive to avoid unnecessary neuroimaging in low-risk patients, reducing the risk of radiation exposure and radiation-related disease, while delivering cost-effective medical care in the setting of rising healthcare costs [13,14]. There is also considerable carbon footprint attributed to CT scans [15]. Efforts have been made to standardize and increase the efficiency of head CT usage by the formulation of clinical decision rules such as the Canadian CT Head Rule (CCHR), NEXUS Head CT Instrument, and New Orleans Criteria with varying degrees of adoption and success [16,17,18].

More recently, blood-based biomarkers, such as S100B protein, ubiquitin C-terminal hydrolase-L1 (UCH-L1) and glial fibrillary acidic protein (GFAP) have emerged as potential adjuncts to reduce the need for head CT in low-risk patients [19]. UCH-L1 and GFAP are predominantly expressed from neurons and astrocytes, respectively. These two biomarkers have been evaluated in prospective multicentre studies and subsequently received clearance by the US Food and Drug Administration in March 2023 for evaluation of patients with acute mTBI [20,21,22]. The French Society of Emergency Medicine incorporated the use of S100B (within 3 h of injury) and UCH-L1/GFAP (within 12 h of injury) in their guideline for management of head injuries [23]. However, there is a paucity of data from Asia on the use of these biomarkers in the management of mTBI.

The aim of this study is to validate the performance of the Abbott Alinity i TBI test, which uses UCH-L1 and GFAP at predetermined values in patients with mTBI and GCS 15 to rule out the presence of intracranial injury in a Singapore emergency department. The secondary aim was to compare the biomarker performance with the commonly used CCHR.

## 2. Materials and Methods

We conducted an observational study at the ED of the National University Hospital, Singapore, from 2 December 2024 to 19 March 2025. The National University Hospital is a 1289-bed tertiary academic medical center with an annual ED attendance of about 120,000 patients and is part of the western healthcare cluster in Singapore. Written informed consent was obtained from all patients prior to enrolment. The study protocol was approved by the National Healthcare Group’s Domain Specific Review Board (reference number: 2023/00714). All research was performed in accordance with relevant regulations and in compliance with the Declaration of Helsinki. This prospective observational study is reported according to the Standards for Reporting Diagnostic Accuracy (STARD) reporting guidelines [24]. This study was not prospectively registered in a clinical trial registry.

The inclusion criteria were as follows: patients aged 21 years and above who sustained head injury within 12 h of ED attendance, had a GCS of 15 and required a head CT as per attending physician. This age cut-off was selected as individuals under the age of 21 years are minors under Singapore law, and are thus considered a vulnerable population who cannot legally give informed consent for themselves. Additionally, only patients with GCS 15 were included as those with mTBI GCS 13 and 14 are considered vulnerable due to the assumed cognitive impairment and because they are compromised in their ability to make decisions in their best interests; therefore, it is mandated by the institutional review board to require further safeguards and specific informed consent processes involving legally acceptable representatives.

Study investigators assessed the decisional capacity of fully alert patients for informed consent in accordance with the local institutional review board guidance. Patients who were unable to provide written informed consent and had sustained head injury more than 12 h prior to ED attendance were excluded from the study. Upon obtaining written informed consent from the participants, a one-time 4 mL blood draw into a lithium heparin tube was performed. The tube was then centrifuged (for 10 min at 4 °C at 2100 relative centrifugal force with 9 acceleration and 9 deceleration) within 10 min of blood collection and extracted plasma was stored in 1.5 mL microtubes (Eppendorf, Hamburg, Germany) at −20 °C for analyses in 3 batches. Plasma UCH-L1 and GFAP levels were analyzed at the hospital central laboratory using the Alinity i TBI (Abbott Laboratories, Chicago, IL, USA). Convenience sampling was employed due to the availability to the study investigators; all eligible patients who attended the ED during office hours and when study team members were on shift after office hours were approached for written informed consent.

The Alinity i TBI test is a panel of in vitro diagnostic chemiluminescent microparticle immunoassay which enables quantitative analysis of UCH-L1 and GFAP in human plasma and allows semi-quantitative interpretation of results using the Alinity system. The results of UCH-L1 and GFAP levels were interpreted at predetermined threshold cut-off values of 400 pg/mL and 35 pg/mL, respectively. The UCH-L1 and GFAP results were combined with automatic interpretation into a single biomarker test result (the result was negative only if both biomarkers were below the respective cut-off values) and compared to head CT imaging results (reference standard). Results of the Alinity i TBI test were not provided to the attending clinicians in real time.

Head CT results were taken from the final verified reports generated with the current standard radiology reporting practices by board-certified attending radiologists, who were unaware of study enrollment. No centralized adjudication or formal secondary independent radiology review was performed. Results of the head CT scans were then coded by the blinded investigators as either ‘traumatic lesion present’ or ‘traumatic lesion absent’ based on the final verified radiology report. Chronic or non-traumatic findings were not classified as traumatic lesions for the primary analysis. In the standardized data collection form, traumatic lesions were coded as present if there was a presence of one or more of the following findings on the head CT: hemorrhagic (acute epidural hemorrhage, acute subdural hemorrhage, indeterminate extra-axial hemorrhage, intraventricular hemorrhage, parenchymal hemorrhage, petechial hemorrhage, subarachnoid hemorrhage), non-hemorrhagic (brain edema, brain herniation, non-hemorrhagic contusion, ventricular compression, ventricular trapping) and external or skull (cranial fractures, depressed skull fractures, facial fractures, scalp injury, skull base fractures) injuries. The biomarker test results (positive or negative) were compared with the patient’s CT findings to determine the diagnostic accuracy (sensitivity, specificity, positive and negative predictive values) and the likelihood ratios of the biomarkers.

Relevant patient demographics and characteristics pertaining to the medical history, time of injury, GCS at ED presentation and clinical indications (including criteria for applying the CCHR) for head CT imaging were also collected using a standardized data collection form. Although the CCHR is the most common clinical decision rule used to guide head CT requests, its compliance is variable. Apart from the primary outcome of head CT results, chart reviews were also conducted to evaluate secondary outcomes of residual cognitive impairment, reattendance or readmission up to 90 days after ED or inpatient hospital discharge. Biomarker results were not made available to treating clinicians in real time and did not influence patient management. Accordingly, repeat head CT was not mandated by the study protocol and was performed only if clinically indicated as part of routine care.

### Sample Size Calculation and Statistical Analyses

The clinical sensitivity of the Alinity i TBI test was previously found to be 95.8% [25], and a previous local study showed positive CT brain findings in 14.3% of patients with mTBI [5]. For the estimated prevalence of traumatic lesions on the CT brain of 14.3%, precision of ±0.10, alpha of 5% and expected drop-out rate of 5%, a minimum sample size of 115 patients was required [26].

Data was analyzed using Stata 17 (StataCorp LP, College Station, TX, USA). Categorical variables are presented as proportions and analyzed using the Chi-squared test or Fisher’s exact test, where appropriate. Continuous variables are reported as means with standard deviations or medians with interquartile ranges (IQRs) for normal and skewed distributions, respectively. Dichotomous values of the biomarkers (elevated or not elevated) were correlated with the presence or absence of CT-detected traumatic lesions to determine sensitivity and negative predictive value. Other indicators such as specificity, positive predictive value and likelihood ratios were also ascertained. The 95% confidence intervals around all the measures were calculated using the Wilson score method and Miettinen-Nurminen score method (for likelihood ratios). Statistical significance was set at *p* < 0.05.

## 3. Results

**Figure 1:**
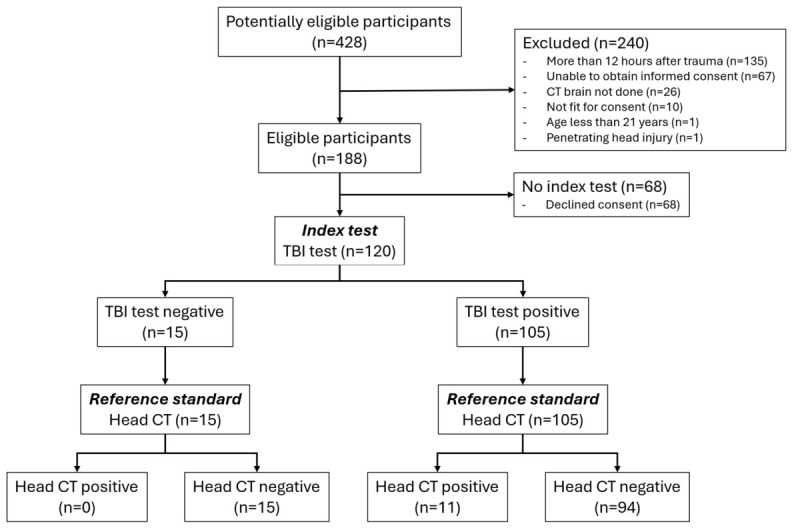
Study flowchart. Abbreviations: CT, computed tomography; TBI, traumatic brain injury.

Between 2 December 2024 and 19 March 2025, 428 adult patients were screened for eligibility to be included in the study (Figure 1). A total of 120 subjects who underwent head CT for mTBI and GCS 15 were ultimately enrolled and included in the final analysis. Overall, there was a predominance of male patients (55.8%, 67/120) and of Chinese ethnicity (75.8%, 91/120) with a median age of 73 (IQR 56 to 79) years (Table 1). There were no differences in age, gender and ethnicity between patients with and without intracranial hemorrhage (ICH) (Table 1). Majority of patients sustained mTBI from falls (81.7%, 98/120) and the second most common mechanism of injury was road traffic incident (10.8%, 13/120). Overall, 9.2% (11/120) of patients had positive head CT findings.

**Figure 2:**
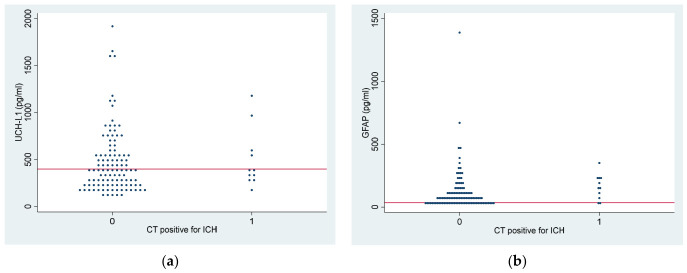
Distribution of UCH-L1 (**a**) and GFAP (**b**) levels and their cut-off values compared to head CT findings. Red lines refer to the cut-off values of 400 pg/ml for UCH-L1 and 35 pg/ml for GFAP. Abbreviations: CT, computed tomography; GFAP, glial fibrillary acidic protein; ICH, intracranial hemorrhage; UCH-L1, ubiquitin carboxyl-terminal hydrolase L1.

A significant proportion of patients with ICH presented with loss of consciousness [63.6% (7/11) versus 25.7% (28/109) in patients with no ICH, *p* = 0.01] and more of these patients had post-traumatic amnesia [27.3% (3/11) versus 7.3% (8/109) in patients with no ICH, *p* = 0.06] (Table 1). Patients with ICH had significantly higher median GFAP levels compared to those without [148.6 (IQR 61.1 to 237.9) pg/mL versus 64.1 (IQR 39.2 to 122.7) pg/mL; *p* = 0.02]; although this difference was not seen in the median UCH-L1 levels between the two groups (Table 1) (Figure 2). Notably, the median UCH-L1 level among CT-negative patients was 381 pg/mL, close to the assay cut-off of 400 pg/mL, with the upper quartile extending above the threshold. This suggests that a substantial proportion (45.0%, 49/109) of CT-negative patients had elevated UCH-L1 levels, which may have contributed to the high false-positive rate and low specificity of the combined test. There were also no differences in proportions of antiplatelet and anticoagulant use among those with ICH and without (Table 1).

The majority (51.7%, 62/120) of patients were admitted to the general ward, while 39.2% (47/120) were discharged directly from the ED. There were 5 (4.2%) patients who were admitted to the ED observational unit, 3 (2.5%) patients discharged against medical advice, and 2 (1.7%) patients were transferred to the operating theater for non-head injury-related surgeries. Only 1 (0.8%) patient was admitted to the neurosurgical high dependency unit for management. There were 2 deaths (both with positive Alinity i TBI tests) unrelated to the index head injury that occurred 53 days and 87 days later.

Four (3.6%) patients (aged 75 to 90 years), all with positive Alinity i TBI tests but negative head CTs, were diagnosed with new cognitive impairment upon evaluation at the outpatient clinic during their follow-up post-mTBI. Twenty-seven (24.5%) patients who had negative head CT reattended the ED after their index head injury, but only 4 of them presented with symptoms related to the mTBI 4 to 27 days later.

A total of 39 patients fulfilled the entry criteria for consideration of applying the CCHR. This yielded 7 patients with true positive results, 26 false positives, 5 true negatives, and 1 false negative. The diagnostic performance of the Alinity i TBI test for the entire cohort, patients below 65 years, and patients fulfilling CCHR criteria are summarized in Table 2. Exploratory post hoc analyses were conducted to assess the diagnostic performance of individual biomarkers of the TBI test, i.e., GFAP and UCH-L1, at their respective predetermined thresholds of 35 pg/mL and 400 pg/mL (Table 2).

An additional comparison was performed between patients recruited during office hours (*n* = 72) and after office hours (*n* = 48). No statistically significant differences were identified between the two groups in age, mechanism of injury, CT positivity rate, or other baseline characteristics examined (Supplementary Table S1).

## 4. Discussion

In this single-center observational study, the Alinity i TBI panel demonstrated superior sensitivity and a negative predictive value of 100% across the entire cohort with mTBI and GCS 15. This is consistent with findings from large validation studies performed in the USA and Europe, reaffirming the reliability of these biomarkers in ruling out intracranial injuries in mTBI patients presenting to the ED within 12 h of injury [20,27,28]. The potential benefits of avoiding unnecessary CT scans are multifold, including reduced patient exposure to ionizing radiation, improved ED resource utilization, and lowered overall healthcare costs. Reduction in carbon emissions can also be achieved as each CT scan produces an average of 9.2 kg carbon dioxide equivalent emissions compared to just 0.16 kg carbon dioxide equivalent emissions for one Alinity i TBI test [15].

The specificity of the TBI panel in our cohort was much lower at 13.8% versus 36.7% in the ALERT-TBI study [20]. Our patients were considerably older, with a median age of 73 years, compared to 48.9 years in ALERT-TBI. The older age could have accounted for higher median biomarker concentrations of GFAP and UCH-L1 levels at 66.9 pg/mL and 380 pg/mL, respectively observed in our study (versus 24.1 pg/mL and 270.1 pg/mL in ALERT-TBI), despite comparable median time of injury to blood draw (3.5 h versus 3.2 h in ALERT-TBI). Age-related chronic elevations in baseline GFAP and UCH-L1 levels are well recognized and postulated to be due to the astroglial activation and neuronal turnover associated with cerebral small-vessel disease, white matter changes, prior silent infarcts, and neurodegenerative processes [29]. Higher prevalence of prior strokes, leukoaraiosis, and atrophy in the elderly increases the likelihood that biomarker elevations reflect background pathology rather than acute traumatic lesions, which dilutes the discriminative performance of GFAP and UCH-L1 for acute findings [30,31]. Age-related renal impairment and systemic inflammation may prolong biomarker persistence, widening the window of detectable elevation after minor insults or non-traumatic brain conditions, further lowering specificity [32]. In our cohort, the low specificity observed appears to have been driven in part by UCH-L1 elevations among patients with negative head CT findings. As the combined Alinity i TBI result is positive when either GFAP or UCH-L1 exceeds its threshold, even a modest non-specific elevation in one biomarker can substantially reduce overall specificity.

An additional observation of interest was that four patients with positive Alinity i TBI tests, but negative head CT findings were subsequently diagnosed with new cognitive impairment during follow-up. Although the numbers are very small and no causal inference can be made, this observation raises the possibility that some biomarker-positive/CT-negative cases may represent an injury not captured by routine CT, or alternatively non-specific biomarker elevation. We have framed this as hypothesis-generating only.

The sensitivity and the negative predictive value remained high in the older population, indicating that a negative result has some clinical utility for ruling out acute intracranial lesions. However, many clinical rules already recommend head CT for older patients (e.g., age ≥ 65 with anticoagulation or concerning mechanism) [33]. In these scenarios, biomarkers add limited efficiency because the imaging would be obtained regardless of these recommendations. The main clinical benefit for older patients is therefore in the carefully selected, lower-risk presentations where a negative biomarker panel can support shared decision-making to avoid an immediate head CT. Conversely, a positive biomarker in older adults should be interpreted cautiously, recognizing the higher false-positive rate from chronic brain changes.

Considering recent evidence that suggested GFAP alone was able to enhance the specificity without diminishing sensitivity, we conducted a post hoc analysis of individual biomarker components of the Alinity i TBI test [34,35]. There was modest improvement in specificity from 13.8% to 21.1% from GFAP alone (Table 2). GFAP is largely astrocyte-specific and predominantly derived from the central nervous system, so elevations could more directly reflect brain parenchymal injury or blood–brain barrier disruption compared to UCH-L1, which is also expressed in peripheral neurons [36]. UCH-L1 tends to rise and peak very rapidly after injury and can be influenced by the short-lived, non-specific insults [37]. It may also be affected by renal function and other comorbidities, leading to transient elevations unrelated to intracranial pathology. GFAP’s release and clearance profile may be less susceptible to these non-specific fluctuations, reducing false positives [36,37].

Younger patients are particularly at risk of the detrimental effects of ionizing radiation as a typical head CT delivers approximately 1.6 mSv of radiation, which is equivalent to 7 months of natural background radiation exposure. In our cohort, 23.1% (9/39) of patients aged below 65 years underwent unnecessary head CT. A Greek study recently attempted to improve the specificity of the panel by defining new cut-offs of 115 ng/L for GFAP and 335 ng/L for UCH-L1 in mTBI patients older than 65 years [38]. We explored the proposed cut-offs in our cohort. Among 89 patients who were 65 years and older, 1 (1.1%) would have had a false negative TBI test with a corresponding decrease in sensitivity (87.5%) despite a modest increase in specificity (34.2%). Another single-center Croatian study stratified cut-offs based on 3 age bands [39]. However, the inclusion of nasal bone fractures, paranasal sinus hemorrhage, subdural hygroma and periorbital hematoma as positive CT findings greatly limits the validity of their findings.

Despite including only patients with a GCS of 15 (versus GCS 14-15 in ALERT-TBI) with a relatively lower impact mechanism of injury (82% simple falls vs. 51.0% in ALERT-TBI), our study had a higher incidence of intracranial injury (9%) compared to ALERT-TBI (6%). Patients with full GCS are expected to have less likelihood of traumatic intracranial injury compared to those with decreased GCS [40]; likewise, patients who sustain lower-impact mechanisms have a lower likelihood [41]. The older age of our cohort and a high prevalence of antiplatelet and/or anticoagulant use (47%) likely contributed to this unexpected finding. Age-related changes in the brain, including brain atrophy, loss of elasticity in the bridging veins, and increased adherence of the dura to the skull, can lead to a higher risk of intracranial bleeding, especially subdural hemorrhage, in older persons [42]. The use of dual antiplatelet agents and/or anticoagulants is also associated with higher bleeding risks, even with seemingly minor injury mechanisms [43,44].

Widely used clinical decision rules (e.g., CCHR, New Orleans Criteria) were not validated in populations taking antiplatelet agents such as clopidogrel and ticagrelor, and no rule reliably identifies a low-risk subset for intracranial hemorrhage in this group [45]. The widely adopted CCHR demonstrated a low sensitivity of 75.0% and a specificity of 38.7% in our local setting; hence, it is not ideal to be used in isolation as a risk stratification tool to reduce head CT use [5]. Multi-dimensional risk assessment, incorporating existing clinical decision rules (considering age, mechanism of injury, clinical symptoms, and signs) and considerations for other specific risk factors such as the use of antiplatelet and/or anticoagulant, combined with biomarker testing could be a synergistic strategy to improve the overall predictive accuracy of intracranial injuries in mTBI patients [46].

### Limitations

The study was conducted at a single tertiary center in Singapore, which may limit the generalizability of results to other settings or populations with differing demographics, injury patterns, and clinical practices. In addition, the age distribution of our cohort is heterogenous, which could influence the patient risk profiles. However, the main bulk of the patients who undergo head CT for mTBI in Singapore are older people with a low-energy mechanism of injury, as represented in our study. Hence, the study results will apply to aging countries with similar patient profiles.

Although additional comparison of patients recruited during office hours and after office hours did not identify significant differences in the variables examined, convenience sampling and clinician-directed CT ordering may still have introduced residual selection and spectrum bias. Additionally, the high number of excluded patients may also have contributed to the selection bias. In particular, patients presenting more than 12 h after injury may differ clinically from those presenting earlier, and exclusion of patients unable to provide written informed consent may have omitted patients with different clinical characteristics. These factors may have influenced the observed sensitivity, and specificity estimates and may limit generalizability. These factors should be considered when interpreting our findings. The reference standard in our study for determining a ‘positive CT’ was the presence of a traumatic brain lesion based on routine board-certified radiologists’ reports, rather than centralized adjudication. This approach may introduce interobserver variability, expectation bias from clinical context, and potential misclassification of traumatic versus chronic or nontraumatic findings.

For informed consent purposes, our study focused exclusively on patients with a GCS of 15, which could introduce selection bias and limit extrapolation to patients with GCS 13–14 that are also currently categorized as mTBI. These patients may have a higher prevalence of intracranial injury and potentially different biomarker performance characteristics. However, TBI patients in the GCS 15 subgroup represent a substantial proportion of patients who present to the ED with a head injury and depict a common subgroup in whom head CT is frequently performed despite relatively low diagnostic yield. This finding was supported by Bazarian et al. in which patients with GCS 15 constituted 94.1% of the mTBI cohort with a comparable incidence of traumatic injury on head CT (5.3%) versus the entire cohort (6.3%) [25]. Demonstrating excellent rule-out performance in this population supports the safe reduction of unnecessary imaging and highlights the test’s clinical utility in low-risk settings.

In our cohort, which was predominantly elderly with a median age of 73 years, age-related renal impairment is likely prevalent and may have contributed to non-specific GFAP and UCH-L1 elevations, further reducing the specificity of the combined test [47]. Although renal function was not systematically assessed in this study, this represents a plausible mechanistic explanation for the false-positive results and underscores the importance of evaluating renal function as a covariate in future biomarker validation studies.

Lastly, although the sensitivity and negative predictive value were both 100% in our cohort, these estimates should be interpreted with caution because only 11 patients had CT-positive lesions. The resulting confidence intervals were wide, reflecting limited precision and reduced stability of these point estimates. Larger validation studies are needed to confirm the robustness of these findings.

## 5. Conclusions

The high rule-out performance of the Alinity i TBI test supports using these biomarkers as a safe tool in patients with mTBI and GCS 15 presenting within 12 h of injury. However, the observed lower specificity undermines its potential for healthcare cost savings associated with the reduction in head CT usage. Exploratory post hoc findings regarding individual biomarkers, particularly GFAP alone, should be interpreted cautiously. Future studies should focus on optimizing specificity while maintaining a reasonably high degree of sensitivity. Other areas of research should also focus on the cost-effectiveness of incorporating biomarker testing in routine mTBI evaluation.

## Figures and Tables

**Table 1:** Baseline demographics, mechanisms of injury, clinical findings and outcomes.

Variables	Total (*n* = 120)	CT Positive for ICH (*n* = 11)	CT Negative for ICH (*n* = 109)	*p* Value
Age (years), median (IQR)	73 (56–79)	76 (55–83)	72 (57–79)	0.48
Patients ≥ 65 years, *n* (%)	81 (67.5)	8 (72.7)	73 (67.0)	1.00
Males, *n* (%)	67 (55.8)	6 (54.6)	61 (56.0)	1.00
Ethnicity, *n* (%)				
Chinese	91 (75.8)	8 (72.7)	83 (76.1)	0.79
Malay	12 (10.0)	1 (9.1)	11 (10.1)	
Indian	13 (10.8)	2 (18.2)	11 (10.1)	
Others	4 (3.3)	0	4 (3.7)	
Mechanism of injury, *n* (%)				
Fall	98 (81.7)	10 (90.9)	88 (80.7)	0.39
Road traffic incident	13 (10.8)	0	13 (11.9)	
Sports	4 (3.3)	0	4 (3.7)	
Struck by object	3 (2.5)	1 (9.1)	2 (1.8)	
Pedestrian struck by vehicle	2 (1.7)	0	2 (1.8)	
Loss of consciousness, *n* (%)	35 (29.2)	7 (63.6)	28 (25.7)	0.01
Post-traumatic amnesia, *n* (%)	11 (9.2)	3 (27.3)	8 (7.3)	0.06
Witnessed disorientation, *n* (%)	1 (0.8)	1 (9.1)	0	0.09
Antiplatelet, *n* (%)	38 (31.7)	5 (45.5)	33 (30.3)	0.32
Anticoagulant, *n* (%)	18 (15.0)	2 (18.2)	16 (14.7)	0.67
Intoxicated with alcohol, *n* (%)	3 (2.5)	0	3 (2.8)	1.00
Time from injury to blood draw (min), median (IQR)	210 (161–355)	210 (190–250)	210 (160–355)	0.87
GFAP (pg/mL), median (IQR)	66.9 (40.4–146.2)	148.6 (61.1–237.9)	64.1 (39.2–122.7)	0.02
UCH-L1 (pg/mL), median (IQR)	380 (232–560)	370 (285–579)	381 (231–555)	0.58
Intracranial head CT findings, *n* (%)				
SAH and SDH		2 (18.2)		
SAH and IPH		2 (18.2)		
SAH		3 (27.3)		
SDH		2 (18.2)		
IPH		2 (18.2)		

Abbreviations: CT, computed tomography; GFAP, glial fibrillary acidic protein; ICH, intracranial hemorrhage; IPH, intraparenchymal hemorrhage; IQR, interquartile range; SAH, subarachnoid hemorrhage; SDH, subdural hemorrhage; UCH-L1, ubiquitin carboxyl-terminal hydrolase L1.

**Table 2:** Diagnostic performance of the Alinity i TBI test for the entire cohort and those below 65 years, GFAP, UCH-L1 and the Canadian Computed Tomography Head Rule for eligible patients.

Variables	All Patients (*n* = 120)	Patients < 65 Years (*n* = 39)	GFAP (Cut-Off 35 pg/mL) (*n* = 120)	UCH-L1 (Cut-Off 400 pg/mL) (*n* = 120)	Patients Fulfilling CCHR (*n* = 39)
Prevalence	9.2 (4.7–15.8)	7.7 (1.6–20.9)	9.2 (4.7–15.8)	9.2 (4.7–15.8)	21.0 (9.3–36.5)
Sensitivity	100 (71.5–100)	100 (29.2–100)	100 (71.5–100)	36.4 (10.9–69.2)	87.5 (47.3–99.7)
Specificity	13.8 (7.9–21.7)	25.0 (12.1–42.2)	21.1 (13.9–30.0)	55 (45.2–64.6)	16.1 (5.5–33.7)
Positive predictive value	10.5 (5.4–18.0)	10.0 (2.1–26.5)	11.3 (5.8–19.4)	7.55 (2.09–18.2)	21.2 (9.0–38.9)
Negative predictive value	100 (78.2–100)	100 (66.4–100)	100 (85.2–100)	89.6 (79.7–95.7)	83.3 (35.9–99.6)
Likelihood ratio positive test	1.16 (1.08–1.25)	1.33 (1.1–1.61)	1.27 (1.15–1.4)	0.81 (0.36–1.82)	1.04 (0.77–1.41)
Likelihood ratio negative test	0	0	0	1.16 (0.72–1.86)	0.78 (0.11–5.73)

Data reported as % (95% confidence interval). Abbreviation: CCHR, Canadian computed tomography head rule; GFAP, glial fibrillary acidic protein; UCH-L1, ubiquitin carboxyl-terminal hydrolase L1.

## Data Availability

The data presented in this study are available on request from the corresponding author. The data are not publicly available due to restrictions by the approving institutional review board.

## Supplementary Materials

The following supporting information can be downloaded at: https://www.mdpi.com/article/10.3390/medicina62061095/s1. Supplementary Table S1. Comparison of patients recruited during and after office hours.

## Abbreviations

The following abbreviations are used in this manuscript:

TBI	Traumatic brain injury
ED	Emergency department
GCS	Glasgow Coma Scale
mTBI	Mild traumatic brain injury
CT	Computed tomography
CCHR	Canadian Computed Tomography Head Rule
UCH-L1	Ubiquitin C-terminal hydrolase-L1
GFAP	Glial fibrillary acidic protein
STARD	Standards for Reporting Diagnostic Accuracy
IQR	Interquartile range
ICH	Intracranial hemorrhage
IPH	Intraparenchymal hemorrhage
SAH	Subarachnoid hemorrhage
SDH	Subdural hemorrhage
