# Supplementary material for: Validation of Blood-Based Biomarkers After Mild Traumatic Brain Injury with GCS 15 in a Singapore Emergency Department: An Observational Study
Source: Medicina (Kaunas). 2026 Jun 5;62(6):1095. doi: 10.3390/medicina62061095 (PMC13302830; doi:10.3390/medicina62061095)
Supplement: Supplementary file 1 [file medicina-62-01095-s001.zip › medicina-4324892-supplementary.pdf]

Supplementary Table S1. Comparison of patients recruited during and after office hours.

Variables	Total (*n* = 120)	During office hours (*n* = 72)	After office hours (*n* = 48)	*p* Value
Age (years), median (IQR)	73 (56–79)	72 (60–79)	74 (54.5–78.5)	0.86*
Males, n (%)	67 (55.8)	43 (59.7)	24 (50.0)	0.29
Ethnicity, n (%)				
Chinese	91 (75.8)	59 (81.9)	32 (66.7)	0.29
Malay	12 (10.0)	5 (6.9)	7 (14.6)	
Indian	13 (10.8)	6 (8.3)	7 (14.6)	
Others	4 (3.3)	2 (2.8)	2 (4.2)	
Mechanism of injury, n (%)				
Fall	98 (81.7)	61 (84.7)	37 (77.1)	0.10
Road traffic incident	13 (10.8)	7 (9.7)	6 (12.5)	
Sports	4 (3.3)	0	4 (8.3)	
Struck by object	3 (2.5)	2 (2.8)	1 (2.1)	
Pedestrian struck by vehicle	2 (1.7)	2 (2.8)	0	
Loss of consciousness, n (%)	35 (29.2)	21 (29.2)	14 (29.2)	1.00
Post-traumatic amnesia, n (%)	11 (9.2)	6 (8.3)	5 (10.4)	0.70
Witnessed disorientation, n (%)	1 (0.8)	0	1 (2.1)	0.40^
Antiplatelet, n (%)	38 (31.7)	25 (34.7)	13 (27.1)	0.53
Anticoagulant, n (%)	18 (15.0)	10 (13.9)	8 (16.7)	0.90
Intoxicated with alcohol, n (%)	3 (2.5)	0	3 (6.3)	0.06^
Reattendance to the ED, n (%)	27 (22.5)	18 (25.0)	9 (18.8)	0.42
GFAP (pg/ml), median (IQR)	66.9 (40.4–146.2)	72.9 (40.8–163.6)	59.5 (35.8–118.0)	0.16*
UCH-L1 (pg/ml), median (IQR)	380 (232–560)	394 (227–672)	326 (224–521)	0.23*
	**(*n* = 11)**	**(*n* = 4)**	**(*n* = 7)**	
Intracranial head CT findings, n (%)				
SAH and SDH	2 (18.2)	1 (25.0)	1 (14.3)	0.29^
SAH and IPH	2 (18.2)	1 (25.0)	1 (14.3)	
SAH	3 (27.3)	0	3 (42.8)	
SDH	2 (18.2)	1 (25.0)	1 (14.3)	
IPH	2 (18.2)	1 (25.0)	1 (14.3)	

Abbreviations: CT, computed tomography; ED, emergency department; GFAP, glial fibrillary acidic protein; ICH, intracranial hemorrhage; IPH, intraparenchymal hemorrhage; IQR, interquartile range; SAH, subarachnoid hemorrhage; SDH, subdural hemorrhage; UCH-L1, ubiquitin carboxyl-terminal hydrolase L1.

All *p* Values obtained by chi-squared test unless otherwise stated. * Wilcoxon rank-sum test. ^ Fisher's exact test.
